# Potential Use of Turkish Medicinal Plants in the Treatment of Various Diseases

**DOI:** 10.3390/molecules21030257

**Published:** 2016-02-25

**Authors:** Gulay Ozkan, Senem Kamiloglu, Tugba Ozdal, Dilek Boyacioglu, Esra Capanoglu

**Affiliations:** 1 Department of Food Engineering, Faculty of Chemical and Metallurgical Engineering, Istanbul Technical University, Maslak, 34469 Istanbul, Turkey; ozkangula@itu.edu.tr (G.O.); skamiloglu@itu.edu.tr (S.K.); boyaci@itu.edu.tr (D.B.); 2 Department of Food Engineering, Faculty of Engineering and Architecture, Okan University, Akfirat-Tuzla, 34959 Istanbul, Turkey; tugba.ozdal@okan.edu.tr; 3 Scientific Bio Solutions LLC., Maslak, Istanbul, Turkey

**Keywords:** medicinal plants, antioxidant activity, cancer, cardiovascular diseases, diabetes, infectious, wound treatment, arthritis, neurological disorders, gastric disorders

## Abstract

Medicinal plants are sources of health-promoting substances, including phytochemicals and phytoalexins that comprise polyphenols, flavonoids, carotenoids, vitamins A, C, E and several other constituents. Many studies have indicated that medicinal plants have been used to treat human diseases for thousands of years owing to their antimicrobial and antioxidant activities. Medicinal plants reduce the oxidative stress in cells and prevent cancer, cardiovascular and inflammatory diseases, neurodegenerative and digestive system disorders. These potential beneficial effects have been attributed to the presence of bioactive compounds that show antioxidant properties by acting as free radical scavengers or metal chelators, reducing the reactions that produce reactive oxygen and nitrogen species (ROS/RNS). Considering the importance of medicinal plants in terms of their beneficial health effects, some of the medicinally important plants grown in Turkey are covered in this review with respect to their antioxidant potential and phytochemical profile.

## 1. Introduction

A great number of fruits, vegetables, aromatic, spicy, medicinal and other plants may contain bioactive compounds exhibiting free radical scavenging activity. Many medicinal plants include large amounts of antioxidants such as phenolic compounds, nitrogen compounds, vitamins, terpenoids and other endogenous metabolites [1,2,3,4,5]. Indeed, from ancient times to modern times, plant-based systems have been used in many areas, including nutrition, medicine, flavoring, beverages, cosmetics, *etc.* [6,7]. According to the World Health Organization, approximately 80% of the world’s population in developing countries relies on traditional medicines, which are mostly derived from plants, for their primary health care [6]. Epidemiological studies have shown that many of the phytochemicals from medicinal plants possess anti-inflammatory, antiatherosclerotic, antitumor, antimutagenic, anticarcinogenic, antibacterial, or antiviral activities [8,9]. They are also associated with reduced risks of cancer, cardiovascular disease, diabetes and lower mortality rates of several human diseases [10,11,12].

In Turkish folk medicine, different parts of the plant species are used to prepare ethnomedicines. Aerial parts, leaves, fruits, seeds, flowers, roots and bulbs are the most frequently used parts of the medicinal plants. In some cases, other ingredients including sugar, honey, alcohol or flour are also used to prepare the remedies [13]. Moreover, several formulations such as herbal teas, extracts, decoctions, infusions, tinctures, *etc.* are the methods mostly used for the preparation of the folk medicines [14]. Herbal teas are mixtures of unground or suitably ground medicinal plants, which may include medicinal plant extracts, ethereal oils or medicinal substances. Extracts are prepared by extracting plants with suitable extraction agents. The extract obtained after separation of the liquid from the plant residue is used as an intermediary product, which is to be further processed as quickly as possible. Decoctions are prepared by soaking the plants in the water at a temperature above 90 °C for 30 min with repeated stirring, continued with straining while the mixture is still hot. Infusions are prepared mixing the plants in a mortar with water and allowing the mixtures to stand for 15 min. After adding boiling water to the mixture and 5 min of repeated stirring, the suspension is left to stand until it is cooled. Tinctures are extracts from medicinal plants of varying concentrations prepared with ethanol [15].

In the present review, the antioxidant properties of medicinal plants, which are used in Turkish folk medicine, are evaluated in terms of their bioactive compounds. Furthermore, the association between some medicinal plants and prevention of diseases including cancer, cardiovascular diseases, diabetes, infections, wound treatment, arthritis, neurological and gastric disorders are highlighted.

## 2. Antioxidant Properties of Medicinal Plants

**Figure 1:**
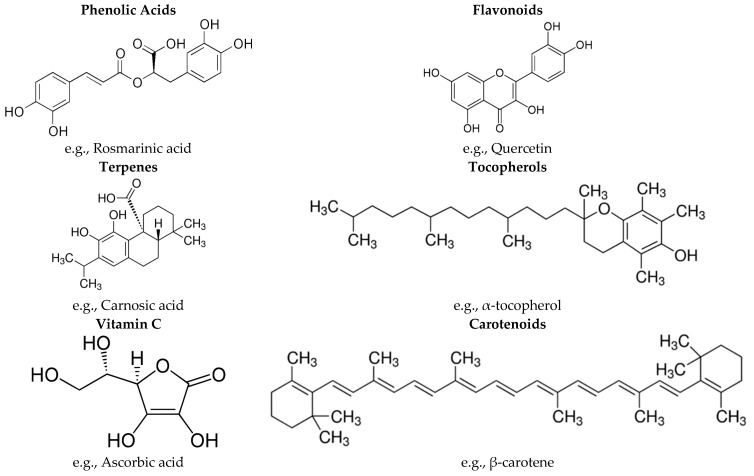
Major antioxidants in medicinal plants.

Many constituents of medicinal plants may contribute to the antioxidant and other related protective properties. Among these compounds phenolic acids, flavonoids, terpenes, tocopherols, vitamin C and carotenoids merit special consideration as they are well distributed in several medicinal plants. These antioxidants in medicinal plants may act independently or possess combined synergistic effect [16]. Examples of some common antioxidants present in medicinal plants are given in Figure 1.

Among the phenolic acids, rosmarinic acid (Figure 1) is the most predominant phenolic compound in many medicinal plants especially of Lamiaceae family. It was identified as the major component in basil (*Ocimum basilicum* L.), oregano (*Origanum vulgare* L.), rosemary (*Rosmarinus officinalis* L.), sage (*Salvia officinalis* L.), savory (*Satureja hortensis* L.) and thyme (*Thymus vulgaris* L.), and was present in the range of 36.3 to 145.0 mg/g. [17]. Protocatechuic, *p*-coumaric, chlorogenic, ferulic, and caffeic acids are some other phenolic acids present in medicinal plants [18], including linden (*Tilia argentea*) (5.8–7924.0 μg/g dw), hawthorn (*Crataegi folium*) (4.6–3566.6 μg/g dw) and bistort (*Polygonum bistorta*) (4.4–3049.9 μg/g dw) [19].

Recently, flavonoids have gained great interest as potential therapeutic agents against a wide variety of diseases, most of which involve radical damage. Flavonoids can interfere not only with the propagation reactions of the free radical, but also with the formation of the radicals [20]. The most common flavonoids present in medicinal plants include quercetin (Figure 1), kaempferol, luteolin and apigenin [21,22]. The amount of these flavonoids in *Helichrysum chasmolycicum* P.H. Davis ranged from 3.0 to 15.0 mg/g [21], whereas in sage (*Salvia fructicosa* Miller) they were present in the range of 0.5 to 1.2 mg/g [22].

The major terpenes present in medicinal plants are mono- and diterpenes. Monoterpenes (C_10_), composed of two C_5_ isoprene units, which allows for a wide variety of structures, constitute 90% of plant essential oils [23]. Carvacrol, menthol, myrcene and thymol are among the most widespread monoterpenes present in medicinal plants including oregano (*Origanum onites* L.) (0.1%–65.5%), sage (*Salvia triloba* L.) (1.1%–3.5%), mint (*Mentha piperita* L.) (0.1%–35.1%), and laurel (*Laurus nobilus* L.) (0.2%–0.3%) [24]. The most common diterpene in medicinal plants is carnosic acid (Figure 1), which has a similar structure to rosmarinic acid. The oxidative hydroxylation of carnosic acid leads to the formation of carnosol, a derivative with increased stability, while still possessing antioxidant properties. The recovery of carnosol in marjoram (*Origanum majorana* L.) was between 24.0%–37.0% of the raw material [25].

Tocopherols control the accumulation of reactive oxygen species in plastids, playing a major role in controlling singlet oxygen levels within thylakoid membranes. α-Tocopherol (Figure 1) supplies protection to membranes mainly by quenching singlet oxygen and reacting with lipid peroxy radicals and has been shown to reduce the extent of lipid peroxidation in leaves and seeds [26]. Tocopherols are present in medicinal plants including fennel (*Foeniculum vulgare*) (44.0 μg/g dw), cumin (*Cuminum cyminum* L.) (54.0 μg/g dw), and caraway (*Carum carvi* L.) (58.0 μg/g dw) [27,28,29].

Vitamin C (Figure 1) is present in some fresh medicinal plants such as mint (*Mentha piperita* L.) (52.6 mg/100 g), lemon balm (*Melissa officinalis* L.) (53.2 mg/100 g), and oregano (*Origanum onites* L.) (23.1 mg/100 g) [30]. During food processing, vitamin C can easily be degraded, depending on many variables such as pH, temperature, light, and the presence of enzymes, oxygen, and transition metal ion catalyzers. In fact, drying has been shown to decrease the vitamin C content significantly (by up to 90%) [16,31].

Carotenoids, particularly β-carotene (Figure 1), lutein, and zeaxanthin are present in some fresh medicinal plant such as basil (*Ocimum basilicum*) (25.8 mg/100 g), coriander (*Coriandrum sativum*) (14.4 mg/100 g), dill (*Anethum graveolens*) (8.7 mg/100 g), mint (*Mentha piperita* L.) (9.0 mg/100 g), parsley (*Petroselinum crispum*) (6.6 mg/100 g), rosemary (*Rosmarinus officinalis*) (2.1 mg/100 g), sage (*Salvia officinalis*) (2.0 mg/100 g), and tarragon (*Artemisia dracunculus* L.) (11.1 mg/100 g) [32]. Similar to ascorbic acid, drying decreases the carotenoid content in plants (around 50%) [16]. Effects of all these antioxidants present in Turkish medicinal plants on several diseases including cancer, cardiovascular diseases, diabetes, infectious diseases, and other diseases are covered in this review.

## 3. Cancer

Numerous physiological and biochemical processes including ultraviolet radiation, tobacco smoke [33], infections by virus [34], bacteria [35] and parasites [36], contamination of foods by mycotoxins [37], free radicals, and reactive oxygen species [38,39] lead to chronic diseases such as cancer. Cancer is the second leading cause of death after cardiovascular diseases [40]. Globally, the number of cancer deaths is estimated to increase from 7.1 million in 2002 to 11.5 million in 2030 [41]. It has been an important case to prevent and to find an effective drug to treat cancer [42]. However, the conventional cancer treatments -chemotherapy and radiotherapy- are expensive and have many side effects, including vomiting, alopecia, diarrhea, constipation, myelosuppression, neurological, cardiac, pulmonary, and renal toxicity [43]. Therefore, there is a need to discover alternative anticancer drugs which are more selective and less toxic than those currently in use [44].

During the 1960s, the U.S. National Cancer Institute started to screen about 35,000 plant samples from 20 countries and examined 114,000 extracts for anticancer activity [45]. At this time, more than 3000 plants worldwide have been reported to have anticancer properties [46]. Approximately, 60% of drugs currently used for cancer treatment have been isolated from natural products [47]. Different medicinal plants have been investigated and used for their anticancer properties worldwide. Similary, in Turkish folk medicine different medicinal plants have been reported to be used for the prevention of cancer as listed in Table 1.

Ozkan and Erdoğan [48] investigated the antioxidant and anticancer properties of essential oil from Origanum onites and its two major phenolic components, carvacrol, and thymol. Their cytoprotective effects against hydrogen peroxide and membrane damage in hepatoma G2 (Hep G2) cells were examined. Antioxidant properties were determined by means of DPPH radical scavenging activity and linoleic acid oxidation inhibition. The DPPH radical scavenging activity (EC_50_: 80 μg/mL) of the essential oil was found to be higher than carvacrol and thymol. The linoleic acid oxidation inhibition rate of the essential oil (40%) was close to its two major components. The maximum protective concentration of essential oil on Hep G2 cells was found to be 20 μg/mL, whereas carvacrol and thymol reached to the maximum cytotoxic effects with 10.62 μg/mL and 24 μg/mL, respectively. According to the cytotoxic effect results, the essential oil (IC_50_: 149.12 μg/mL) was found to be less toxic than carvacrol (IC_50_: 53.09 μg/mL) and thymol (IC_50_: 60.01 μg/mL) on Hep *G2* cells.

Moreover, the viability of the Hep G2 cells decreased with increasing concentrations (20–170 μg/mL) of essential oil, carvacrol and thymol, and did not change at concentrations of 170 μg/mL or higher. Additionally, the essential oil showed a higher membrane-protective effect than thymol and carvacrol. It was concluded that essential oil obtained from *O. onites* and its two major components exhibit antioxidant and carcinogenesis-reducing potential [48].

Ozmen *et al.* [50] studied the anti-leukemic effect of *Scutellaria orientalis*. The strongest anti-leukemic activity was detected in the methanolic extract (IC_50_ of 43 mg/mL) among petroleum ether, dichloromethane, and ethyl acetate. It was also found that methanolic extract contained apigenin, baicalein, chrysin, luteolin, and wogonin with strong anti-proliferative activity [50].

Similarly, anti-leukemic effects of triterpene saponins from *Astragalus* species (*Astragalus brachypterus, Astragalus cephalotes, Astragalus microcephalus*, and *Astragalus trojanus*) and methanolic extracts from the roots of three Astragalus species (*Astragalus cephalotes, Astragalus oleifolius* and *Astragalus trojanus*) were investigated. Cycloartane- and oleanan-type triterpenes from these species possess prominent IL-2 inducing activities which contribute anticancer effects of Astragalus species. It was demonstrated that all triterpene saponins showed a prominent IL-2 inducing activity changing between 35.9% and 139.6%. Among the extracts the highest value was obtained for *Astragalus oleifolius* (141.2%) [49].

Even though a variety of medicinal plants are used for their anticancer properties in Turkish folk medicine, more *in vitro* and *in vivo* studies should be carried out to be sure of this effect. On the other hand, possible toxic effects and toxic doses should also be considered.

## 4. Cardiovascular Diseases

Cardiovascular disease (CVD) is a multifactorial disorder with a high mortality rate which is the leading cause of death worldwide. Out of the 16 million deaths under the age of 70 due to non-communicable diseases, 82% are in low and middle income countries and 37% are caused by CVDs [96]. The World Health Organization (WHO) estimated that 17.5 million people died from CVDs in 2012, representing 31% of all global deaths. Of these deaths, an estimated 7.4 million were due to coronary heart disease and 6.7 million were due to stroke. WHO also estimated that over three quarters of CVD deaths take place in low- and middle-income countries [97].

Epidemiological studies have revealed that high consumption of plant foods reduce the risk of CVDs, but the role of bioactive compounds providing these protective effects is still under investigation [98,99,100,101]. Clinical and preclinical studies also supported that plant foods that contain high content of flavonoids or isolated flavonoids demonstrate beneficial effects on cardiovascular risk factors such as blood pressure, endothelial function, platelet function, and cholesterolemia [102,103,104,105,106,107,108,109,110]. On the other hand, ethnomedicinal survey studies show that medicinal plants are used traditionally as a protective natural medicine in Turkey [111,112,113,114,115,116].

Angiotensin converting enzyme (ACE) is a carboxydipeptidase related to renin-angiotensin system and kinin nitric oxide system. In renin-angiotensin system, ACE cleaves angiotensin I to a potent vasoconstrictor angiotensin II. Besides, ACE inactivates the hypotensive peptide bradykinin in the kinin nitric oxide system [117]. ACE plays an important role in the regulation of blood pressure and normal cardiovascular function. Catalysis of angiotensin I to angiotensin II causes increase in blood pressure, and therefore inhibition of ACE may help in the management of hypertension [118]. In Turkey, ACE inhibitory activity of traditionally consumed medicinal plants has also been reported (Table 1).

Dalar and Konczak [52] studied the suppression of ACE of six traditional medicinal tea (*Verbascum cheiranthifolium, Anchonium elrichrysifolium, Plantago lanceolata, Phlomis armeniaca, Phlomis armeniaca, Malva neglecta, Salvia limbata*) infusions. They have observed the highest ACE inhibitory activity displayed by *S. limbata* (35.0%) and *M. neglecta* (34.0%) herbal infusions at the concentration of 0.6 mg/mL. The remaining herbal infusions exhibited lower effect with 21%–27% enzyme inhibition. Captopril, an ACE inhibitor, is currently used in the treatment of hypertension and was used as a positive control in this study. ACE inhibitory drugs are widely used, however they possess some adverse health effects when used over prolonged time [119]. Natural sources are the potential alternatives to the synthetic ACE inhibitors. Among the evaluated herbal infusions, *S. limbata* and *M. neglecta* exhibited the strongest and comparable inhibitory activity against ACE and the highest captopril equivalent [52].

In another study by the same group in 2014, the suppression of ACE by *Cichorium intybus* L. was investigated. They have observed that the ACE inhibitory activity of *C. intybus* L. extract equaled 1.5 ± 0.2 µmol/g dw captopril equivalents and at the final concentration of 0.6 mg/mL it inhibited enzyme activity by 21.0% ± 2.0% [51].

Considering the rich variety of medicinal plants commonly grown and used in Turkey and the high prevalence of cardiovascular diseases, more investigations should be carried out in order to understand the effects of Turkish medicinal plants on protecting cardiovascular diseases. On the other hand, there are limited number of studies in the literature related with the ACE inhibitory activity of Turkish medicinal plants, so the ACE inhibitory activity of these traditionally consumed medicinal plants should also be studied. Besides, mechanism of action of the phytochemicals in these medicinal plants are not fully known, therefore more detailed studies are required.

## 5. Diabetes

Diabetes mellitus is a major endocrine disorder, affecting approximately 5% of the world’s population. WHO estimates that almost 3 million deaths occurring annually are as a result of diabetes and that there will be 366 million cases of diabetes by the year 2030 [120]. Diabetes is characterized by abnormalities in carbohydrate, lipid and lipoprotein metabolisms, which not only lead to hyperglycaemia but also cause many complications such as hyperlipidemia, hyperinsulinemia, hypertension and atherosclerosis [121,122].

There is increasing evidence that complications associated with diabetes may be related to oxidative stress induced by the production of free radicals [57]. Pancreatic β-cells are particularly susceptible to the detrimental effects of reactive oxygen species (ROS), because of their low expression of the antioxidant enzymes genes as compared to other tissues. Thus, the increase of ROS leads to damage of β-cells through the induction of apoptosis and suppression of insulin biosynthesis [121]. Antioxidants have been shown to prevent the destruction of β-cells by inhibiting the peroxidation chain reaction and thus they may provide protection against the development of diabetes [54].

Although the pharmacological treatment of diabetes mellitus is based on oral hypoglycemic agents and insulin injection [123], in many countries, various antioxidant plants are used traditionally as an alternative strategy for the treatment of diabetes. With the active encouragement of the WHO, an attempt is being made to collect traditional medical information used for the treatment of diabetes for study in modern laboratories in order to scientifically evaluate therapeutic efficacies [54]. In Turkish folk medicine, some traditional and edible plants have also been utilized to decrease symptoms of diabetes. Among these plants, the ones listed in Table 1 have been studied recently.

Diabetes mellitus has generally been induced to laboratory animals by injecting drugs such as streptozotocin (STZ). Animals treated with STZ also have increased hepatic and renal malondialdehyde levels and decreased levels of hepatically reduced glutathione (GSH) concentration [124]. *In vivo* reports on antidiabetic properties of medicinal plants generally measure biomarkers such as blood glucose level and compare the parameters with healthy animals. Aslan *et al.* [54] showed that in STZ-induced diabetic rats oral administration of *Cydonia oblonga* leaf (500 mg/kg) and *Allium porrum* bulb (500 mg/kg) extracts for 5 days caused a decrease in blood glucose levels by 34% and 18%, respectively. Moreover, *Helianthus tuberosus* tuber and *A. porrum* extracts showed an inhibitory effect on kidney tissue thiobarbituric acid reactive substance (TBARS) levels by 25% and 15%, suggesting that these extracts have protective effect against lipid peroxides produced by diabetes [54].

Sezik *et al.* [56] investigated the hypoglycaemic and antioxidant effects of the *Helichrysum plicatum* plant extract in STZ-induced diabetic rats for 15 days during pregnancy. The extract (250 mg/kg) led to decreased blood glucose (24% and 29%), increased serum insulin, and decreased serum triglycerides in both pregnant and non-pregnant diabetic animals. Liver TBARS and reduced GSH measurements in extract-treated diabetics were similar to non-diabetic pregnant controls, indicating probable reversal of increased lipid peroxidation in the liver [56]. Similarly, Orhan *et al.* [59] determined the hypoglycaemic and antidiabetic activities of *Juniperus oxycedrus* leaves in glucose-hyperglycemic and STZ-induced diabetic rats. The extract has shown long term inhibitory effect on blood glucose levels of diabetic rats [59]. Furthermore, in another study conducted by the same group [58] extracts of *Cistus laurifolius* (250–500 mg/kg) have been shown to decrease the blood glucose levels of the STZ-induced diabetic rats as compared to control group (16%–34%). For glucose loaded rats, *C. laurifolius* extracts have shown a weak hypoglycemic effect (11%–20%) [58].

Ozkol *et al.* [57] studied the therapeutic potential of *Urtica dioica, Thymus vulgaris, Myrtus communis, Scolymus hispanicus* and *Cinnamomun zeylanicum* (100 mg/kg for each) on STZ-induced diabetic rats for 28 days. The results showed that only *S. hispanicus* extract significantly amended fasting blood glucose level, whereas all plant extracts increased reduced GSH content and decreased lipid peroxidation levels of erythrocyte, plasma, retina and lens tissues. Moreover, none of the plant extracts counteracted body weight loss of diabetic rats [57]. Likewise, Bayramoglu *et al.* [61] evaluated the protective effect of carvacrol isolated from essential oil of *Origanum onites* (25–50 mg/kg) in STZ-induced diabetic rats for a period of 7 days. Although treatment of diabetic rats with oral administration of carvacrol resulted in a slight reduction in serum glucose level and significant reduction in serum total cholesterol, alanine aminotransferase, aspartate aminotransferase and lactate dehydrogenase in comparison with diabetic control rats, there were no significant differences in serum insulin levels and body weight changes. Despite the inadequacy of carvacrol on diabetes treatments, it was determined to have a partially protective role on liver enzymes [61]. More recently, Dogan *et al.* [60] demonstrated that extract from *Quercus brantii* exerts certain antidiabetic properties in the small intestine of STZ-induced diabetic rats. Diabetic serum biomarkers such as fasting glucose level, glycosylated hemoglobin and α-glycosidase activity were significantly increased in diabetic group as compared to control group whereas, the serum insulin level significantly decreased. In addition, oral administration of *Q. brantii* extract for 21 days restored the parameter values to near normal in diabetic rats [60].

α-Amylase and α-glucosidase are known as key enzymes in starch breakdown and intestinal absorption. Inhibition of these enzymes can effectively control of blood glucose levels in diabetes mellitus [66]. Therefore, plant extracts exhibiting a low α-amylase and a high α-glucosidase inhibitory activity are preferred in the treatment or prevention of diabetes [53]. The activity of plant extracts against these enzymes is generally expressed as an IC_50_ value, which corresponds to a half maximal inhibitory concentration. An *in vitro* study on the essential oil of *Eucalyptus camaldulensis* leaves indicated that the α-amylase and α-glucosidase were inhibited (IC_50_: 0.435 and 0.548 μL/mL, respectively) by a non-competitive mechanism [65]. Same authors later studied the α-glucosidase inhibitory activity of essential oil of *Laurus nobilis* and concluded that the essential oil inhibits the α-glucosidase competitively [64]. Dalar and Konczak [52] investigated some traditional herbal infusions from Eastern Anatolia region of Turkey and reported that *Phlomis armeniaca, Salvia limbata* and *Plantago lanceolata* exhibits weak inhibitory activities against α-amylase and pronounced inhibitory activities against α-glucosidase, which suggests potential antidiabetic properties [52]. In other studies carried out by the same group, the authors demonstrated that the leaf extracts of *Cichorium intybus* and *Centaurea karduchorum*, medicinal plants endemic to Eastern Anatolia, Turkey, also exhibited a marked inhibitory activity towards α-glucosidase (IC_50_: 4.25 and 0.63 mg/mL, respectively) and a weak inhibitory activity towards amylase (IC_50_: 18.3 and 14.6 mg/mL, respectively), which suggests a potential to reduce postprandial hyperglycaemia and supports their traditional use as antidiabetic agents [51,53].

Orhan *et al.* [58] found that *Cistus laurifolius* may be a potent inhibitor of α-glucosidase and α-amylase, possibly due to polyphenols present in the extract. Similarly, same group reported that the extracts of *Helichrysum graveolens, Juniperus communis* and *Juniperus oxycedrus* exhibit antidiabetic effects with *J. communis* showing the highest α-glucosidase inhibitory activity (IC_50_: 4.4 μg/mL) [55]. Zengin *et al.* [67] showed that *Haplophyllum myrtifolium* possessed a good inhibitory activity on α-glucosidase and α-amylase (up to 0.73 and 2.30 mmol acarbose equivalent/g extract, respectively). Later, same group also studied the essential oil from *Sideritis galatica* that exhibited a marked inhibitory activity on α-glucosidase with an IC_50_ of 0.632 mg/mL, which may be related to monoterpene hydrocarbon content [63]. In similar studies, essential oils from *Origanum vulgare* [62] and the extracts of *Potentilla anatolica* [66] also exhibited α-amylase and α-glucosidase inhibitory activities (up to 0.14–2.10 and 6.04–54.85 mmol acarbose equivalent/g extract, respectively). Still more research is necessary to understand the exact mechanism of the components present in medicinal plants in reducing the risk of diabetes.

## 6. Infectious Diseases

Infectious diseases are caused by pathogenic microorganisms, such as bacteria, viruses, parasites or fungi; the diseases can be spread, directly or indirectly, from one person to another. Turkey has about 75 million inhabitants and 1.67% of them died from infectious diseases [125]. The most common bacterial infections in Tukey are brucellosis, tularaemia, tuberculosis, lyme disease, and rickettsioses. Besides, in Turkey people have more prevalence to viral infectious diseases such as human immunodeficiency virus (HIV), Crimean-Congo haemorrhagic fever, hantavirus infections, West Nile virus (WNV) infection and hepatitis A-D viruses [126].

High antibiotic resistance rates in Turkey have been linked to excessive antibiotic consumption [127]. Antibiotic resistance is the major clinical problem for the treatment of infectious diseases. Considering the side effects of synthetic drugs, medicinal plants were preferred as it was believed that they have less or no side effects. Besides, medicinal plants were economical, effective and relatively less toxic [128]. In Turkey, few medicinal plants were studied for their role in the treatment of infectious diseases (Table 1).

Ozbilgin *et al.* [69], studied the *in vivo* antimalarial effect of medicinal plants that have been traditionally used in Turkey including *Lavandula stoecheas* subsp. cariensis, *Phlomis nissolii, Phlomis bourgaei, Phlomis leucophracta, Centaurea hierapolitana, Centaurea polyclada, Centaurea lydia, Scrophularia cryptophila, Scrophularia depauperata, Scrophularia floribunda, Rubia davisiana* and *Alkanna tinctoria* subsp. subleiocarpa. They have given 250 to 500 mg/kg doses of plant extracts to mice as a single daily dose for 4 days. They have observed that *P. nissolii* water extract, *C. lydia* chloroform extract, *S. cryptophila* ethanol extract, and *C. polyclada* methanol extract showed antimalarial activity with reducing parasitaemia. They have detected the chemotherapeutic effects of plant extracts ranged between 13.5% and 66.9%. Besides, they have observed the chemosuppressions exerted by combined plant extracts of *P. nissolii, S. cryptophila,* and *C. lydia* with *C. polyclada* methanol extract as 51.25%, 57.33 %, and 58.33 %, respectively [69]. Tekeli *et al.* [70] studied the antibacterial effects of twelve *Centaurea* species of which five are endemic to Turkey flora on four infectious bacteria (*Escherichia coli, Bacillus cereus, Salmonella enteritidis, Staphylococcus aureus*). They have observed that *C. cariensis* subsp. microlepis exhibited an antimicrobial effect on all tested microorganisms. Besides, they stated that the extracts from eight *Centaurea* species (C. *balsamita*, *C. calolepis*, *C. cariensis subsp. maculiceps, C. cariensis* subsp. *microlepis, C. kotschyi* var. kotschyi, *C. solstitialis* subsp. solstitialis, *C. urvillei* subsp. urvillei and *C. virgata*) possessed antibacterial activity against several of the tested microorganisms [70].

Furthermore, Albayrak and Aksoy [68] studied the antibacterial effect of methanol extracts of *Anthemis fumariifolia* and *Anthemis cretica* subsp. argaea, which are commonly used in Turkish folk medicine against 13 bacteria and two yeasts. Their results dedicated that the tested medicinal plant extracts had great potential of antibacterial activity against many pathogenic bacteria tested. However, they had no inhibitory effect on *Candida albicans* and *Saccharomyces cerevisiae* [68].

Kozan *et al.* [72] studied the *in vivo* anthelminthic efficacy of 13 *Verbascum* species growing in Turkey, including *V. chionophyllum* Hub.-Mor., *V. cilicicum* Boiss., *V. dudleyanum* (Hub.-Mor.) Hub.-Mor., *V. lasianthum* Boiss., *V. latisepalum* Hub.-Mor., *V. mucronatum* Lam., *V. olympicum* Boiss., *V. pterocalycinum* var. mutense Hub.-Mor., *V. pycnostachyum* Boiss. & Heldr., *V. salviifolium* Boiss., *V. splendidum* Boiss., *V. stachydifolium* Boiss. & Heldr. and *V. uschackense* (Murb.) Hub.-Mor. Their study revealed that the extracts from *V. lasianthum*, *V. latisepalum*, *V. mucronatum* and *V. salviifolum* showed the highest inhibitory rates against *Aspiculuris tetraptera* at 100 mg/kg concentrations in mice. Additionally, they have found that extracts from *V. dudleyanum* and *V. pterocalycinum* var. mutense are generally highly effective [72].

Tuberculosis is an infectious bacterial disease caused by *Mycobacterium tuberculosis*, which most commonly affects the lungs. It is a well-known disease that has afflicted humans since ancient times. Although tremendous efforts have been made to control tuberculosis, the development of drug resistance, multidrug-resistant (MDR) and extensively drug resistant (XDR) strains present significant threats to tuberclosis control. The alarming increase of cases requires the urgent development of new, more effective and safer anti-tuberculosis (anti-TB) drugs. Askun *et al.* [71] studied the antituberclosis effect of six Turkish medicinal plants of the family Lamiaceae (*Stachys tmolea* Boiss., *Stachys thirkei* C. Koch, *Ballota acetabulosa* (L.) Benth., *Thymus sipthorpii* Benth., *Satureja aintabensis* P.H. Davis, and *Micromeria juliana* (L.) Benth. ex Reich.). Their study revealed that *S. aintabensis*, *T. sibthorpii*, and *M. juliana* develop considerable activity against the four strains of *M. tuberculosis* with the minimal inhibitory concentrations value of 12.5–100 μg/mL. *S. aintabensis* and *T. sibthorpii* extracts inhibited *M. tuberculosis* with the minimum bactericidal concentration value of 50–800 μg/mL. According to their results, it can be concluded that these medicinal plants can be used as a source of natural anti-tuberculosis drugs [71].

In Turkey, a variety of medicinal plants are used against infectious diseases. However, their phytochemistry has not been screened and their pharmacological effects against infectious bacteria, viruses, parasites and fungi, which could support their use as a traditional natural medicine is not clarified. Moreover, bio-assay guided fractionation to isolate and identify the active compounds to understand the mechanism of inhibition of pathogenic microorganisms is also required.

## 7. Other Diseases

### 7.1. Wound Healing

Wound is defined as physical, chemical, thermal, microbial or immunological injuries that result with the disruption of the cellular continuity, anatomy, and function of a tissue [129]. Wounds have an impact on a large number of patients and reduce their quality of life [130]. They affect both physical, mental health of patients and charge significant cost on them. Current estimates indicate that nearly 6 million people suffer from chronic wounds worldwide [131]. Wounds produce inflammatory mediators that cause pain and swelling. They are a source for infection and may lead to organ failure or death of the patient [132].

Therefore, proper healing of wounds is essential for the remedy of disrupted cellular continuity, anatomy and function of the tissue. Wound healing is a complex process that involves inflammatory phase (0–3 days), proliferative phase (3–12 days) and remodeling phase (3–6 months) [133,134]. Upon injury to the tissue, a set of collaborative efforts including platelet aggregation, formation of fibrin clot, inflammatory response, angiogenesis, and re-epithelialization take place to repair the damage [135,136]. The aim of healing of wound is to shorten the time required for healing and to minimize the undesired consequences [137]. Wound healing in the human body is affected by many factors such as diet, infection at the wound site, tissue perfusion to the wound area, drugs, age, diabetes, and other disease conditions [138].

Medical treatment of wounds includes local or systemic drugs in an attempt to aid wound repair [139]. On the other hand, it has been reported that many medicinal plants possess healing activity and used to treat wounds. In the present study, in order to elucidate the traditional use of these plants from the scientific point of view, mostly consumed plants in Turkish folk medicine for wound healing were evaluated (Table 1).

The leaves of *Sambucus ebulus* are used in Turkish folk medicine for treatment of rheumatic pains, snake bites, and wounds. Süntar *et al.* [73] investigated the wound healing activity *in vivo* from four different solvent extracts prepared by *n*-hexane, diethyl ether, ethyl acetate, and methanol extractions using these leaves. The wound healing activity of these extracts was studied by means of pharmacological methods in mice and rats. Histopathological examination was also performed in the tissue sections of the wound area. Faster re-modeling was observed in the extract treated groups in comparison to the vehicle and negative control groups. The size of the wound areas and percentage contraction rates were evaluated for 12 days. In the animals treated with the methanolic extract at 1% concentration, maximum healing effect was observed starting from day 6 with 47.6% and reached to 85.4% contraction on day 10 among other extracts. A flavonoid derivative of quercetin 3-O-glucoside was isolated as an active component of the methanolic extract. Additionally, a significant increase with 43.7% in the wound tensile strength was detected with the same extract [73].

In another study conducted by Süntar *et al.* [75], oily extract of *Hypericum perforatum* (10 g plant/100 mL olive oil) was assessed in terms of wound healing activity by *in vivo* excision and incision wound models. In linear incision wound model, two linear skin incisions are created, while a circular wound is created in excision wound model. Results showed that olive oil extract of *H. perforatum* has a remarkable wound healing effect on excision (5.1%–82.6% inhibition) and circular incision (20.2%–100.0% inhibition) wound models. Flavonoids (hyperoside, isoquercitrin, rutin, and (−)-epicatechin) and naphthoquinones (hypericins) were also identified as the bioactive components of *H. perforatum* [75].

*In vivo* wound healing activities of three endemic *Ononis* species including *Ononis sessilifolia*, *Ononis basiadnata* and *Ononis macrosperma* were assessed by histopathological analysis. The aqueous and ethanolic extracts of the aerial parts of *O. macrosperma* (200 mg/kg) exhibited the highest wound healing activity with 35.7% and 37.6%, respectively. Moreover, phenolic compounds and flavonoids were found to be the main active components of antioxidant, anti-inflammatory and wound healing activities of Ononis species [74].

Similarly, Akdemir *et al.* [76] investigated the wound healing activity of *Verbascum mucronatum* by using similar healing experimental models including incision and excision models on mice and rats. Results showed that aqueous extract of this plant displayed significant wound healing activity on linear incision and excision wound model whereas chloroform and methanol extracts were not active. It was also reported that the contraction values of wounds on excision wound model for aqueous extract and its fraction were found to be 30.22% and 46.84% on day 10 and 12, which were compared to the reference drug madecassol (72.24%–100%). Active fractions of ajugol, aucubin, lasianthoside, catalpol, ilwensisaponin A and C, and verbascoside may be responsible from the anti-inflammatory, antinociceptive and wound healing activities [76]. Unfortunately, there are limited studies on the wound healing effect of Turkish medicinal plants. Further *in vivo* and *in vitro* studies should be performed to understand the effective traditional medicinal plants in order to understand their potential to be used as a wound healing agent.

### 7.2. Rheumatoid Arthritis

Rheumatoid arthritis (RA) is a chronic, systemic inflammatory disorder of many tissues and organs. The disorder is characterized by inflammation of the flexible (synovial) membranes of the joints [140]. The disease may produce only mild to moderate symptoms in some patients, whereas in most cases, it lead to hot, painful and swollen joint as well as reducing the function and mobility if not adequately treated [141]. RA occurs at any age but is more common in the middle age [123]. About 0.5%–1% of the world’s population suffer from RA, with approximately three times as many women affected as men [142].

Substantial amount of works in this field have been performed, however, the causes of RA are currently not well understood. It is generally accepted to be an autoimmune disorder, which may be triggered by environmental conditions in genetically susceptible individuals [143].

Current RA treatment strategies aim to decrease the pain and swelling symptoms by using anti-inflammatory agents and to modify the disease process by using anti-rheumatic drugs. However, because of side effects and toxicity, these current treatments are not ideal for prolonged usage [141]. Therefore, there is a need to reveal safer and less toxic alternatives for the treatment of RA and to assess the potential of some medicinal plants for curing or preventing the disease [144]. Some of the medicinal plants which are used in Turkey for the treatment of rheumatoid arthritis have been listed in Table 1.

Çadırcı *et al.* [78] investigated water, methanol, *n*-butanol, acetone, and chloroform extracts from three *Salvia* species (*S. fruticosa, S. verticillata*, and *S. trichoclada*) to screen their anti-inflammatory activity by using *in vivo* carrageenan-induced inflammatory paw odema model. Carrageenan-induced inflammation effects of the extracts were found to be the highest after 3 h. Anti-inflammatory effects of these extracts were found to be in the range of 3.57%–48.21%. It was also obtained that n-butanol extract of *Salvia fruticosa* (100 mg/kg) was the highest with 48.21% anti-inflammatory effect. These results were comparable with a reference drug of indomethacin (25 mg/kg) with 66.07% inhibition effect. It is obvious that the polar extract of *Salvia fruticosa* provides pharmacological support to folkloric uses of Turkish sage in the treatment of anti-inflammatory disorders. Moreover, the active flavonoids, phenolic acids and terpenoids were reported to be the responsible components for the anti-inflammatory activity of these plants [78].

Similarly, Akdemir *et al.* [76] evaluated the anti-inflammatory activity of methanol, chloroform and aqueous extracts from *Verbascum mucronatum* by using carrageenan-induced inflammatory paw odema model. The aqueous extract (200 mg/kg) provided significant anti-inflammatory inhibition ranging between 15.9% and 30.7% against carrageenan-induced hind paw odema model and the results were comparable to a reference compound of indomethacin (35.1%–41.1% inhibition) [76]. On the other hand, Türel *et al.* [79] found significant reduction in paw odema by using *Plantago major*. However, they did not exhibit as strong effect as indomethacin which produced a significant inhibition (90.01%). It was reported that the reduction in the inflammation was 45.87% with the dose of 20 mg/kg and 49.76% with the dose of 25 mg/kg [79].

In another study, acetic acid-induced increase in capillary permeability test was used to assess anti-inflammatory effect of *Centaurea iberica.* A dose-dependent inhibitory activity was observed for methanol extract of *C. iberica* and the highest inhibitory value was found to be 31.6% with the dose of 200 mg/kg [77].

In order to decrease symptoms of RA, medicinal plants, which have anti-inflammatory effect can be used. However, there is limited research to investigate the anti-inflammatory effects of Turkish medicinal plants. On the other hand, the phytochemistry and pharmacology of these plants should be investigated, particularly for any activities associated with the treatment of RA.

### 7.3. Neurological Disorders

Neurodegeneration is a complex procedure causing neuronal death associated with many devastating diseases including Alzheimer′s disease (AD), Parkinson′s disease (PD), prion diseases and amyotrophic lateral sclerosis (ALS). Among them, AD is a progressive neurological disorder, the most common type of dementia, which is characterized by the memory and behavioral dysfunctions particularly in elderly population. Some mechanisms have been proposed for pathogenesis of the disease, which are metal dysregulation leading to oxidative damage, deficit in acetylcholine level as well as aggregation of toxic amyloid fibrils on amyloid beta (Aβ) peptide [86]. The cholinergic hypothesis is the most accepted theory to explain pathogenesis of AD, and therefore the most prescribed drugs for the treatment of AD are the cholinesterase inhibitors [80]. These inhibitors promote an increase in the level of acetylcholine in neuronal synaptic area, which is considered to play a vital role in the memory disturbances of AD patients [62]. Some synthetic compounds such as tacrine and galantamine are used as cholinesterase inhibitors for treatment of neurological disorders. However, the short half-lifes and the health risks associated with the toxicity of these drugs (hepatotoxicity, gastrointestinal disturbances, *etc.*) have resulted in an ongoing research for naturally occurring acetylcholinesterase (AChE) and butyrylcholinesterase (BChE) inhibitors, especially of plant origin [82,84]. In Turkey, cholinesterase inhibitory activity of traditionally consumed medicinal plants has also been studied (Table 1).

In most of the *in vitro* studies cholinesterase inhibitory activity was examined using Ellman’s colorimetric method. In this method, generally less than 50% inhibition of AChE is not considered significant. The studies reveled that extracts prepared with different solvents show varying AChE inhibitory activity. For instance, in the study of Akkol *et al.* [87] and Demirzezer *et al.* [89] ethanol extract of *Cistus laurifolius* leaf and methanol extract of *Salvia trichoclada* exerted the highest AChE inhibition (approximately 80%), respectively. Similarly, chloroform extract of *Arnebia densiflora* roots [85], ethyl acetate extract of *Calendula arvensis* flowers [81], ethyl acetate and methanol extracts of *Viburnum tinus* [80], acetone extract of *Ballota nigra* [88], petroleum ether extracts of *Haplophyllum myrtifolium* [67], water and methanol extracts of *Alpinia officinarum* [92], *n*-hexane/dichloromethane extract of *Onosma nigricaule* roots [86] and ethyl acetate extract of *Potentilla anatolica* [66] were the most active in AChE inhibition (31%–98%). Extracts from *Centaurea polypodiifolia* [82] and essential oils from *Origanum vulgare* [62] and *Sideritis galatica* [63] had a noticeable inhibition towards both AChE and BChE, suggesting that these species could be utilized as anticholinesterase agents. On the other hand, *Galium spurium* extract exhibited 16% inhibition against BChE, whereas it did not inhibit AChE [91]. Likewise, *Myrtus communis* [90] fruit extract, *Centella asiatica* [83] and *Hypericum capitatum* [86] were active towards BChE.

Demirezer *et al.* [89] carried out an *ex vivo* study on isolated guinea pig ileum to determine the possible inhibitory effects of *Salvia* extracts on AChE. The volatile oil of *Salvia trichoclada* completely blocked the acetylcholine-induced contraction in isolated guinea pig ileum.

Recent *in vivo* studies on the possible effect of medicinal plants cultivated in Turkey on neurological disorders is quite limited, possibly due to ethical concerns. However, in order to confirm the results obtained *in vitro*, further *in vivo* investigations needs to be done.

### 7.4. Gastric Disorders

Ulcers are an open sore of the skin or mucus membrane characterized by sloughing of inflamed dead tissue and a common gastrointestinal disorder worldwide. It is basically an inflamed break in the skin or the mucus membrane lining the alimentary tract [145]. Peptic ulcer is one of the world’s major gastrointestinal disorders and affecting 10% of the world population [146]. Ulcers are commonly present on skin of the lower extremities and in the gastrointestinal tract. There are many types of ulcer including mouth ulcer, esophagus ulcer, peptic ulcer, and genital ulcer, but peptic ulcer is the most widely seen ulcer type. The peptic ulcers are erosion of lining of stomach or the duodenum [147]. The two most common types of peptic ulcer are called “gastric ulcer” and “duodenal ulcer.” Although patients with gastric ulcers have normal or decreased acid production, ulcers may occur even in complete absence of acid [148].

The pathophysiology of peptic ulcer disease involves an imbalance between offensive (acid, pepsin, and *Helicobacter pylori*) and defensive factors (mucin, prostaglandin, bicarbonate, nitric oxide, and growth factors) [149]. A number of synthetic drugs are available to treat ulcers. But these drugs are expensive and are likely to produce side effects. In Turkey various medicinal plants are used traditionally to cure ulcers as they are economical and as they are believed to have less or no side effects (Table 1).

Gurbuz *et al.* [93] investigated the anti-ulcerogenic effects of *Styrax liquidus* obtained from *Liquidambar orientalis* and its fractions obtained by successive solvent extractions with chloroform and *n*-butanol against the ethanol-induced peptic ulcer model in rats. They have observed that the chloroform extract demonstrated a statistically significant gastroprotective effect. In addition, the chemical characterization of the volatiles obtained by microdistillation technique from *Styrax liquidus* and the sub-extracts were analyzed by gas chromatography (GC) and gas chromatography–mass spectrometry (GC–MS), respectively. Their experiments have clearly demonstrated that 150 and 300 mg/kg doses of *Styrax liquidus* given orally to rats showed significant gastric protection. On GC–MS analysis of the resin, overall, 31 compounds representing 99.8% of the total oil were identified where styrene (81.9%), cinnamyl alcohol (6.9%) and α-pinene (3.5%) were identified as the major components. This study confirmed the anti-ulcerogenic activity of the local ethnobotanical usage of *Styrax liquidus* in Turkey [93].

Moreover, Yesilada and Gurbuz [94] studied the bioactivity of 3-*O*-1’’’-β-d-glucopyranoside isolated from the aerial parts of *Equisetum palustre* L. used traditionally to treat peptic ulcer disease in Turkey by successive chromatographical methods. The activity profile of the compound was investigated using several *in vivo* ulcerogenesis models such as indomethacin-, indomethacin plus HCl/EtOH-, cysteamine-, serotonin-, NG-nitro-l-arginine methyl ester plus EtOH-, diethyldithio-carbamate-, *N*-ethylmaleimide plus EtOH-, water immersion and restraint stress-, pyloric ligation-induced ulcers. In addition, they studied the effects of 3-*O*-1’’’-β-d-glucopyranoside on the biochemical parameters of gastric juice as inhibition of titratable gastric acidity, acid output, gastric pH, gastric secretion volume and peptic activity were studied. According to the results of this study, 3-*O*-1’’’-β-d-glucopyranoside showed statistically significant gastroprotective activity against indomethacin-, indomethacin plus HCl/EtOH- and *N*-ethylmaleimide plus EtOH-induced ulcerogenesis. Moreover, 3-*O*-1’’’-β-d-glucopyranoside performed weak activity against NG-nitro-l-arginine methyl ester plus EtOH, water immersion and immobilization-induced stress, pyloric ligation-induced and diethyldithiocarbamate-induced gastric ulcer models. Furthermore, 3-*O*-1’’’-β-d-glucopyranoside was ineffective in the prevention of ulcers induced by serotonin and cysteamine. Besides, 3-*O*-1’’’-β-d-glucopyranoside was only found to increase the gastric acid pH from 2.03 to 3.35 among the gastric biochemical parameters studied. As a conclusion of this study, it was clearly observed that a flavonol diglucoside named 3-*O*-1’’’-β-d-glucopyranoside extracted and isolated from the Turkish medicinal plant *Equisetum palustre* L. was found to improve the cytoprotective mechanisms of the gastric mucosa while showing a weak activity profile on the parameters affecting the gastric acidity such as water immersion and restraint-induced-, pyloric ligation- induced-ulcerogenesis and titratable acidity [94].

Besides, Yesilada *et al.* [95] observed the antiulcerogenic activity of *Sambucus ebulus* L. leaves on water immersion and immobilization-induced stress ulcer model in rats. Their study investigated the antiulcerogenic activity profile of *Sambucus ebulus* L. leaves on various *in vivo* peptic ulcer models and gastric biochemical parameters and through bioassay-guided processing to isolate the active constituents and elucidated its structure. According to their study, they have observed that the butanol subextract exerted significant antiulcerogenic activity against water-immersion and immobilization-induced stress ulcer model in rats as the bioassay model among the subextracts obtained by successive solvent extractions from the MeOH extract of the *Sambucus ebulus* L. leaves. They also tested the activity of each fraction/subfraction of this subextract by subjecting it to successive chemical separation techniques (precipitation, column chromatography based on ion-exchange, silica gel and Sephadex) using the same bioassay model. Besides, they have performed more studies on the active subextract by using various *in vivo* test models (ethanol-, serotonin-, pyloric ligation-induced ulcerogenesis) in rats as well as biochemical methods for the evaluation of antiulcerogenic potential. According to their bioassay-guided fractionation procedures, two flavonol glycosides, isorhamnetin-3-*O*-monoglycoside and quercetin-3-*O*-monoglycoside, were elucidated as the active anti-ulcerogenic principles by using ^1^H-, ^13^C-NMR, and FAB-MS techniques. Their study proved the traditional use of medicinal plant *Sambucus ebulus* leaves for the treatment of gastric ailments in Turkish traditional medicine [95]. However, further studies should be performed to understand the most effective traditional medicinal plants on gastric disorders.

## 8. Conclusions

Several studies have been carried out on Turkish medicinal plants to investigate their antioxidant activity and thus their protective capacity against some diseases. The positive health effects provided by these medicinal plants are believed to be as a result of phytochemicals present in these plants or their interactions with other components. This review provides scientific data for the ethnomedicinal features of numerous medicinal plants grown or consumed in Turkey. However, it should be kept in mind that more investigations, especially *in vivo* studies, need to be performed to validate the positive health effects of these medicinal plants. Moreover, there needs to be more research on the antioxidant activities of medicinal plants’ aqueous extracts prepared by decoction and infusion instead of alcoholic extracts which are not suitable for direct consumption. On the other hand, the belief that these medicinal plants do not have any side or toxic effects as they are natural may lead to big problems as it is clear that toxicological effects are directly related with the dose, so toxicological data for the use of these medicinal plants should also be provided when discussing the health effects of these plants.

## Figures and Tables

**Table 1:** Documented therapeutic effects of medicinal plants in Turkey.

Effective on	Family	Scientific Name	Parts Used	Bioactive Compounds	Antioxidant Activity	Ref.
Cancer	Fabaceae	*Astragalus brachypterus*	Root	Saponins	Not specified	[49]
		*Astragalus cephalotes*	Root	Saponins	Not specified	[49]
		*Astragalus microcephalus*	Root	Saponins	Not specified	[49]
		*Astragalus oleifolius*	Root	Saponins	Not specified	[49]
		*Astragalus trojanus*	Root	Saponins	Not specified	[49]
	Labiatae	*Scutellaria orientalis*	Root	Apigenin, Baicalein, Chrysin, Luteolin, Wogonin	Not specified	[50]
	Lamiaceae	*Origanum onites*	Aerial parts	Carvacrol, Thymol, Linalool, Cymene, Terpinen-4-ol, γ-Terpinene	DPPH (IC_50_): 80 µg/mL essential oil DPPH (IC_50_): 248 µg/mL carvacrol DPPH (IC_50_): 163 µg/mL thymol β-carotene bleaching: 40% essential oil β-carotene bleaching: 52% carvacrol β-carotene bleaching: 57% thymol MDA (carvacrol pretreated): 0.4 nmol/mg protein MDA (thymol pretreated): ~0.5 nmol/mg protein MDA (essential oil pretreated): 0.3 nmol/mg protein	[48]
Cardiovascular Diseases	Asteraceae	*Cichorium intybus*	Aerial parts	Cichoric acid, 4-*O*-Caffeoylquinic acid, 5-*O*-Caffeoylquinic acid, Luteolin hexoside, Caftaric acid	FRAP: 82.2–251.6 μmol Fe^2+^ Eq./g dw ORAC: 823.9–1307.7 μmol Trolox Eq./g dw	[51]
	Brassicaceae	*Anchonium elrichrysifolium*	Aerial parts	Phenolics	FRAP: ≈400 μmol Fe^2+^ Eq./g dw ORAC: ≈2250.0 μmol Trolox Eq./g dw	[52]
	Lamiaceae	*Phlomis armeniaca*	Aerial parts	Phenolics	FRAP: ≈900.0 μmol Fe^2+^ Eq./g dw ORAC: ≈3000.0 μmol Trolox Eq./g dw	[52]
		*Salvia limbata*	Aerial parts	Phenolics	FRAP: ≈950.0 μmol Fe^2+^ Eq./g dw ORAC: ≈3500.0 μmol Trolox Eq./g dw	[52]
	Malvaceae	*Malva neglecta*	Aerial parts	Phenolics	FRAP: 390.8 μmol Fe^2+^ Eq./g dw ORAC: 1638.4 μmol Trolox Eq./g dw	[52]
	Plantaginaceae	*Plantago lanceolata*	Aerial parts	Luteolin-7-*O*-glucoside, Rutin, Chlorogenic acid, Quercetin hexoside	FRAP: 1130.8 μmol Fe^2+^ Eq./g dw ORAC: ≈3250.0 μmol Trolox Eq./g dw	[52]
	Scrophulariaceae	*Verbascum cheiranthifolium*	Aerial parts	Phenolics	FRAP: ≈1100 μmol Fe^2+^ Eq./g dw ORAC: 4265.9 μmol Trolox Eq./g dw	[52]
Diabetes	Asteraceae	*Centaurea karduchorum*	Root, stem, leaf, flower	Luteolin glucuronide, Luteolin hexoside, Chlorogenic acid, Apigenin glucuronide	FRAP: 274.0–441.0 μmol Fe^2+^ Eq./g dw ORAC: 930.5–1853.5 μmol Trolox Eq./g dw	[53]
		*Cichorium intybus*	Aerial parts	Cichoric acid, 4-*O*-Caffeoylquinic acid, 5-*O*-Caffeoylquinic acid, Luteolin hexoside, Caftaric acid	FRAP: 82.2–251.6 μmol Fe^2+^ Eq./g dw ORAC: 823.9–1307.7 μmol Trolox Eq./g dw	[51]
		*Helianthus tuberosus*	Tubers	Reducing sugars, Flavonoids, Alkoloids, Saponins, Triterpene steroids	TBARS: 10.5%–24.5% GSH: 2.4%–34.4%	[54]
		*Helichrysum graveolens*	Capitulum	Not specified	ABTS: 2.7%–88.5%	[55]
		*Helichrysum plicatum*	Capitulum	Not specified	TBARS: 1.5–316.0 nmol/g GSH: 86.0–107.0 μmol/g	[56]
		*Scolymus hispanicus*	Aerial parts	Not specified	GSH: 2.5–26.0 nmol/mL MDA: 2.8–15.8 nmol/mL GR: 0.3 U/mL GST: 9.1 U/mL CAT: 84.3 U/mL	[57]
	Cistaceae	*Cistus laurifolius*	Leaves	Apigenin, Dimethoxyapigenin, Methoxyapigenin, Naringenin, Quercitrin, Quercetin, Methoxyquercetin, Dimethoxyquercetin, Dimethoxy-kaempferol, Chlorogenic acid, Gallic acid, Ellagic acid	Not specified	[58]
	Cupressaceae	*Juniperus communis*	Fruit, leaf	Not specified	ABTS: 0.0%–99.5%	[55]
		*Juniperus oxycedrus*	Leaves	Hexadecanoic acid, Methyl linolenate, Methyl hexadecanoate, Methyl linolenate, Linoleic acid, Methyl linolenate, Hexadecane, Hexadecanoic acid, Octadecanoic acid, Tetradecane, Oleic acid, (*E,Z*)-2,4-Heptadienal, Methyl octadecanoate	Not specified	[59]
		*Juniperus oxycedrus*	Fruit, leaf	Not specified	ABTS: 0.0%–97.8%	[55]
	Fagaceae	*Quercus brantii*	Acorn	Not specified	CAT: 30.7–558.0 U/mL SOD: 1578.3–2319.1 U/mL GSH-Px: 33.8–167.1 U/mL GST: 4.1–41.7 U/mL GR: 0.2–1.5 U/mL GSH: 4.9–109.0 mg/mL MDA: 26.7–56.9 nmol/mL	[60]
	Lamiaceae	*Origanum onites*	Essential oil	Carvacrol	Not specified	[61]
		*Origanum vulgare*	Essential oil	Linalool, Thymol, Carvacrol, *p*-Cymene, γ-Terpiene, β-Caryophyllene, α-Terpiene, Borneol, 1-Octen-3-ol, Caryophyllene oxide, α-Thujene, *cis*-Linalol oxide, *trans*-Linalol oxide, α-Pinene, β-Bisabolene, *p*‑Cymene-8-ol, Elemol, α-Terpineol, Camphene, Hotrienol, α-Humulene	DPPH: 0.0–57.2 mg Trolox Eq./g oil ABTS: 9.6–176.4 mg Trolox Eq./g oil FRAP: 17.1–133.3 mg Trolox Eq./g oil CUPRAC: 46.6–222.1 mg Trolox Eq./g oil β-carotene bleaching: 24.0%–99.9% Phosphomolybdenum: 0.8–8.1 mmol Trolox Eq./g oil Metal chelating: 1.3–3.8 mg EDTA Eq./g oil	[62]
		*Phlomis armeniaca*	Aerial parts	Phenolics	FRAP: ≈900.0 μmol Fe^2+^ Eq./g dw ORAC: ≈3000.0 μmol Trolox Eq./g dw	[52]
		*Salvia limbata*	Aerial parts	Phenolics	FRAP: ≈950.0 μmol Fe^2+^ Eq./g dw ORAC: ≈3500.0 μmol Trolox Eq./g dw	[52]
		*Sideritis galatica*	Essential oil	β-Pinene, α-Pinene, β-Caryophyllene, (*Z*)-β-Ocimene, Limonene, Benzyl benzoate, Sabinene, β-Phellandrene, (*E*)-β-Ocimene, Germacrene D, Caryophyllene oxide, Bicyclogermacrene, δ-3-Carene, *p*-Cymene, (*Z*)-β-Farnesene, α-Phellandrene, Terpinolene, Heptanal, α-Humulene, 1-Octen-3-ol, δ-Cardinene, α-Copaene, (*E*)-2-Hexenal	DPPH (IC_50_): 16.5 mg/mL ABTS (IC_50_): 8.5 mg/mL NO (IC_50_): 0.9 mg/mL CUPRAC (EC_50_): 1.1 mg/mL FRAP (EC_50_): 2.1 mg/mL Phosphomolybdenum: 2.6 mg Trolox Eq./g oil Metal chelating: 29.1 mg EDTA Eq./g oil	[63]
		*Thymus vulgaris*	Aerial parts	Not specified	GSH: 2.3–29.4 nmol/mL MDA: 3.0–15.5 nmol/mL GR: 0.3 U/mL GST: 10.0 U/mL CAT: 98.4 U/mL	[57]
	Lauraceae	*Cinnamomun zeylanicum*	Aerial parts	Not specified	GSH: 2.2–26.7 nmol/mL MDA: 2.5–18.2 nmol/mL GR: 0.3 U/mL GST: 9.4 U/mL CAT: 86.2 U/mL	[57]
		*Laurus nobilis*	Essential oil	1-8-Cineole, 1-(*S*)-α-Pinene, *R*-(+)-Limonene, Sabinene, *p*-Cymene, α-Terpinene, 1,4-Terpineole, 2-α-Pinene, γ-Terpinene, Camphene, *trans*-Pinocarveole, α-Terpinolene, 1-Phellandrene, Endobornyl acetate, Pinocarvone, *p*-Ment-1-en-8-ol, l-Linalool, Octahydro-8a-hydroxy-4a-methyl-2(1*H*)-naphthalenone, Geosmin, (2-Methylprop-1-enyl)-cyclohexa-1,3-diene, Benzene, Bicyclo[3.1.1] hep-2-en-2-carboxy aldehyde 6,6-dimethyl, Urea, 5-5-Dimethlcyclopentadiene, 3-Hexane-1-ol, *p*-Ment-1-en-3,8-diol, 5,9,9-Trimethylspiro[3.5]non-5-en-1-one, α-Campholene aldehyde, Izomyrisenole	Hydroxyl (IC_50_): 0.4 μL/mL Superoxide (IC_50_): 0.1 μL/mL Hydrogene peroxide (IC_50_ × 10^4^): 2.4 μL/mL Lipid peroxidation (IC_50_): 0.1 μL/mL DPPH (IC_50_): 0.6 μL/mL	[64]
	Liliaceae	*Allium porrum*	Bulbs	Flavonoids, Triterpenoids, Reducing sugars, Alkoloids, Steroidal saponins	TBARS: 1.2%–44.6% GSH: 7.2%–22.0%	[54]
	Myrtaceae	*Eucalyptus camaldulensis*	Essential oil	*p*-Cymene, 1-8-Cineole, 1-(*S*)-α-Pinene, *R*-(+)-Limonene, 1,4-Terpineole, 1-Phellandrene, α-Terpinene, Bicyclo[3.1.0]hex-2-en-4-methylene-1-(1-methylethyl), γ-Terpiene, *trans*-Pinocarveol, *p*-Ment-1-en-8-ol, α-Thujone, 4-(1-Methylethyliden)-cyclohexanone, α-Methyl-benzenmethanol, 2-α-Pinene, (2-Methylprop-1-enyl)-cyclohexa-1,5-diene, α-Terpinolene, Linalool oxide, l-Linalool, *p*-Ment-1-en-3,8-diol, 6-Methyl-3-(1-methylethyl)-2-cyclo-hexane-1-one, 5-Methyl-2-(1- methyl-ethenyl)-*trans*-cyclohexanone, α-Campholene aldehyde, 2-Methyl-5-(1-methylethenyl) (*R*)-2-cyclohexane-1-one, *trans*-Pinocarvyl acetate, 3-Methyl-2-(2-pentenyl) cyclopentanone, 4-Methoxy-7-methyl-*trans*-oxabicyclo[3.3.0] oct-7-en-2-one, l, 5-Amino-4-cyano-3-(4-ethylaminobutyl) pyrazole	Hydroxyl (IC_50_): 0.3 μL/mL Superoxide (IC_50_): 0.1 μL/mL Hydrogene peroxide (IC_50_ × 10^4^): 5.6 μL/mL Lipid peroxidation (IC_50_): 0.1 μL/mL DPPH (IC_50_): 4.1 μL/mL	[65]
		*Myrtus communis*	Aerial parts	Not specified	GSH: 2.3–26.6 nmol/mL MDA: 2.2–15.8 nmol/mL GR: 0.3 U/mL GST: 9.1 U/mL CAT: 102.5 U/mL	[57]
	Plantaginaceae Rosaceae	*Plantago lanceolata*	Aerial parts	Luteolin-7-*O*-glucoside, Rutin, Chlorogenic acid, Quercetin hexoside	FRAP: 1130.8 μmol Fe^2+^ Eq./g dw ORAC: ≈3250.0 μmol Trolox Eq./g dw	[52]
		*Cydonia oblonga*	Leaves	Flavonoids, Tannins, Triterpene steroids, Reducing sugars, Saponins, Alkoloids	TBARS: 0.7%–45.7% GSH: 11.3%–20.8%	[54]
		*Potentilla anatolica*	Aerial parts	Phenolics, Flavonoids, Saponins, Triterpenoids	DPPH: 302.8–334.7 mg Trolox Eq./g ABTS: 4.9–5.2 mmol Trolox Eq./g Phosphomolybdenum: 3.3–4.8 mmol Trolox Eq./g FRAP: 223.6–233.0 mg Trolox Eq./g CUPRAC: 291.7–340.8 mg Trolox Eq./g Metal chelating: 27.4–32.9 mg EDTA Eq./g	[66]
	Rutaceae	*Haplophyllum myrtifolium*	Aerial parts	Phenolics, Flavonoids, Tannins, Saponins, Flavanols	DPPH: 43.8–84.5 mg Trolox Eq./g ABTS: 129.3–263.5 mg Trolox Eq./g NO: 2.6–7.0 mmol Trolox Eq./g Phosphomolybdenum: 1.7–3.3 mmol Trolox Eq./g Metal chelating: 8.4–41.8 mg EDTA Eq./g FRAP: 0.4–0.7 mmol Trolox Eq./g CUPRAC: 0.5–0.8 mmol Trolox Eq./g	[67]
	Urticaceae	*Urtica dioica*	Aerial parts	Not specified	GSH: 2.2–29.4 nmol/mL MDA: 2.6–19.1 nmol/mL GR: 0.3 U/mL GST: 9.4 U/mL CT: 81.7 U/mL	[57]
Infectious Diseases	Asteraceae	*Anthemis cretica*	Aerial parts	Phenolics, Flavonoids	Phosphomolybdenum: 163.5 mg AA Eq./g β-carotene bleaching: 59.1% DPPH: 92.5%	[68]
		*Anthemis fumariifolia*	Aerial parts	Phenolics, Flavonoids	Phosphomolybdenum: 173.2 mg AA Eq./gA β-carotene bleaching: 55.4% DPPH: 90.7%	[68]
		*Centaurea hierapolitana*	Aerial parts	Tannins	Not specified	[69]
		*Centaurea lydia*	Aerial parts	Terpenoids, Flavonoids	Not specified	[69]
		*Centaurea polyclada*	Aerial parts	Terpenoids, Flavonoids, Tannins	Not specified	[69]
	Boraginaceae Centaurea	*Alkanna tinctoria*	Aerial parts	Tannins	Not specified	[69]
		*Centaurea balsamita*	Aerial parts	Not specified	Not specified	[70]
		*Centaurea calolepis*	Aerial parts	Not specified	Not specified	[70]
		*Centaurea carduiformis*	Aerial parts	Not specified	Not specified	[70]
		*Centaurea cariensis*	Aerial parts	Not specified	Not specified	[70]
		*Centaurea iberica*	Aerial parts	Not specified	Not specified	[70]
		*Centaurea kotschyi*	Aerial parts	Not specified	Not specified	[70]
		*Centaurea pterocaula*	Aerial parts	Not specified	Not specified	[70]
		*Centaurea solstitialis*	Aerial parts	Not specified	Not specified	[70]
		*Centaurea triumfettii*	Aerial parts	Not specified	Not specified	[70]
		*Centaurea urvillei*	Aerial parts	Not specified	Not specified	[70]
		*Centaurea virgate*	Aerial parts	Not specified	Not specified	[70]
	Labiatae	*Lavandula stoecheas*	Aerial parts	Tannins	Not specified	[69]
		*Phlomis bourgaei*	Aerial parts	Tannins	Not specified	[69]
		*Phlomis leucophracta*	Aerial parts	Tannins	Not specified	[69]
		*Phlomis nissolii*	Aerial parts	Terpenoids, Flavonoids, Tannins	Not specified	[69]
	Lamineceae	*Ballota acetabulosa*	Aerial parts	Gallic acid, Chlorogenic acid, Caffeic acid, (−)-Epicatechin, *p*-Coumaric acid, Rosmarinic acid, Naringin, Rutin hydrate, Apigenin-7-glucoside, Oleuropein, (±)-Naringenin, Luteolin	Not specified	[71]
		*Micromeria juliana*	Aerial parts	Gallic acid, Chlorogenic acid, Caffeic acid, Syringic acid, *p*-Coumaric acid, Ferulic acid, Vitexin, Rosmarinic acid, Naringin, Rutin hydrate, Hesperidine, Apigenin-7-glucoside, Oleuropein, Quercetin, (±)-Naringenin, Luteolin	Not specified	[71]
		*Satureja aintabensis*	Aerial parts	Gallic acid, Chlorogenic acid, Caffeic acid, Syringic acid, *p*-Coumaric acid, Ferulic acid, Vitexin, Rosmarinic acid, Naringin, Rutin hydrate, Hesperidine, Apigenin-7-glucoside, Oleuropein, Quercetin, (±)-Naringenin, Luteolin	Not specified	[71]
		*Stachys thirkei*	Aerial parts	Gallic acid, Chlorogenic acid, Caffeic acid, Syringic acid, *p*-Coumaric acid, Vitexin, Rosmarinic acid, Naringin, Rutin hydrate, Apigenin-7-glucoside, (±)-Naringenin, Luteolin	Not specified	[71]
		*Stachys tmolea*	Aerial parts	Gallic acid, Chlorogenic acid, Caffeic acid, *p*-Coumaric acid, Ferulic acid, Vitexin, Naringin, Rutin hydrate, Hesperidine, Apigenin-7-glucoside, Oleuropein, Quercetin, (±)-Naringenin, Luteolin	Not specified	[71]
		*Thymus sipthorpii*	Aerial parts	Gallic acid, Chlorogenic acid, Caffeic acid, Syringic acid, *p*-Coumaric acid, Ferulic acid, Vitexin, Rosmarinic acid, Naringin, Rutin hydrate, Apigenin-7-glucoside, Oleuropein, Quercetin, (±)-Naringenin, Luteolin	Not specified	[71]
	Rubiaceae Scrophulariaceae	*Rubia davisiana*	Aerial parts	Tannins	Not specified	[69]
		*Scrophularia cryptophila*	Aerial parts	Terpenoids, Flavonoids, Tannins	Not specified	[69]
		*Scrophularia epauperata*	Aerial parts	Tannins	Not specified	[69]
		*Scrophularia floribunda*	Aerial parts	Tannins	Not specified	[69]
		*Verbascum cilicicum*	Aerial parts	Iridoid glycosides, Triterpenoid saponins	Not specified	[72]
		*Verbascum dudleyanum*	Aerial parts	Iridoid glycosides, Triterpenoid saponins	Not specified	[72]
		*Verbascum hionophyllum*	Aerial parts	Iridoid glycosides, Triterpenoid saponins	Not specified	[72]
		*Verbascum lasianthum*	Aerial parts	Iridoid glycosides, Triterpenoid saponins	Not specified	[72]
		*Verbascum latisepalum*	Aerial parts	Iridoid glycosides, Triterpenoid saponins	Not specified	[72]
		*Verbascum mucronatum*	Aerial parts	Iridoid glycosides, Triterpenoid saponins	Not specified	[72]
		*Verbascum olympicum*	Aerial parts	Iridoid glycosides, Triterpenoid saponins	Not specified	[72]
		*Verbascum salviifolium*	Aerial parts	Iridoid glycosides, Triterpenoid saponins	Not specified	[72]
		*Verbascum splendidum*	Aerial parts	Iridoid glycosides, Triterpenoid saponins	Not specified	[72]
		*Verbascum stachydifolium*	Aerial parts	Iridoid glycosides, Triterpenoid saponins	Not specified	[72]
		*Verbascum terocalycinum*	Aerial parts	Iridoid glycosides, Triterpenoid saponins	Not specified	[72]
		*Verbascum ycnostachyum*	Aerial parts	Iridoid glycosides, Triterpenoid saponins	Not specified	[72]
Wound healing	Caprifoliceae Fabaceae	*Sambucus ebulus*	Leaves	Quercetin 3-*O*-glucoside	Not specified	[73]
		*Ononis basiadnata*	Aerial parts	Phenolics, Flavonoids	Not specified	[74]
		*Ononis macrosperma*	Aerial parts	Phenolics, Flavonoids	Not specified	[74]
		*Ononis* *sessilifolia*	Aerial parts	Phenolics, Flavonoids	Not specified	[74]
	Hypericaceae	*Hypericum perforatum*	Aerial parts	Hyperoside, Isoquercitrin, Rutin, (−)-epicatechin, Naphthoquinones	Not specified	[75]
	Scrophulariaceae	*Verbascum mucronatum*	Leaves, flowers, whole parts	Ajugol, Aucubin, Lasianthoside, Catalpol, Ilwensisaponin A and C, Verbascoside	Not specified	[76]
Rheumatoid arthritis	Asteraceae Lamiaceae	*Centaurea iberica*	Aerial parts	Sesquiterpene lactones	Not specified	[77]
		*Salvia fruticosa*	Aerial parts	Flavonoids, Phenolic acids, Terpenoids	Not specified	[78]
		*Salvia verticillata*	Aerial parts	Flavonoids, Phenolic acids, Terpenoids	Not specified	[78]
		*Salvia trichoclada*	Aerial parts	Flavonoids, Phenolic acids, Terpenoids	Not specified	[78]
	Plantaginaceae	*Plantago major*	Seed	Caffeic acid	Not specified	[79]
	Scrophulariaceae	*Verbascum mucronatum*	Leaves, flowers, aerial parts	Ajugol, Aucubin, Lasianthoside, Catalpol, Ilwensisaponin A and C, Verbascoside	Not specified	[76]
Neurological disorders	Adoxaceae	*Viburnum tinus*	Branch, leaf, fruit	Phenolics, Flavonoids	DPPH: 7.4%–91.7% DMPD: 3.2%–67.1% Superoxide: 38.4% NO: ≈45.0%–75.0% FRAP: 0.1–3.3 μL/mL Phosphomolybdenum: 0.1–2.7 μL/mL Metal chelating: 22.1%–75.4%	[80]
	Asteraceae	*Calendula arvensis*	Leaf, flower	Phenolics, Flavonoids	FRAP: 41.0–479.0 mg/mL DPPH: 3.8%–52.3% Metal chelating: 0.0%–55.2%	[81]
		*Calendula officinalis*	Leaf, flower	Phenolics, Flavonoids	FRAP: 42.0–325.0 mg/mL DPPH: 2.4%–18.8% Metal chelating: 0.0%–74.3%	[81]
		*Centaurea antalyense*	Aerial parts	Phenolics, Flavonoids, Saponins	Phosphomolybdenum: 248.7–528.6 mg AA Eq./g DPPH: 12.3%–86.1% ABTS: 21.4%–90.7% β-carotene bleaching: ≈40.0%–60.0% Metal chelating: 16.3–73.7 mg EDTA Eq./g CUPRAC: 0.3–1.2 abs FRAP: 0.2–0.9 abs	[82]
		*Centaurea polypodiifolia*	Aerial parts	Phenolics, Flavonoids, Saponins	Phosphomolybdenum: 266.3–549.5 mg AA Eq./g DPPH: 19.4%–93.5% ABTS: 16.3%–93.4% β-carotene bleaching: ≈60.0%–80.0% Metal chelating: 17.0–64.7 mg EDTA Eq./g CUPRAC: 0.3–1.5 abs FRAP: 0.2–12.3 abs	[82]
		*Centaurea pyrrhoblephara*	Aerial parts	Phenolics, Flavonoids, Saponins	Phosphomolybdenum: 226.1–371.3 mg AA Eq./g DPPH: 14.0%–90.1% ABTS: 17.7%–91.1% β-carotene bleaching: ≈45.0%–50.0% Metal chelating: 25.7–73.8 mg EDTA Eq./g CUPRAC: 0.3–1.0 abs FRAP: 0.2–0.8 abs	[82]
		*Centella asiatica*	Aerial parts	*p*-Hydroxy-benzoic acid, Vanillic acid, *p*-Coumaric acid, *p*-Coumaric acid, *trans*-Cinnamic acid	DPPH: 17.0%–32.0% FRAP: ≈0.1–0.4 abs	[83]
		*Crepis foetida*	Flower	Phenolics, Flavonoids, Flavanols, Tannins, Saponins	DPPH: 1.4 mmol Trolox Eq./g ABTS: 0.1 mmol Trolox Eq./g Superoxide: 0.4 mmol Trolox Eq./g NO: 3.4 mmol Trolox Eq./g Hydroxyl: 0.3 mmol Trolox Eq./g Phosphomolybdenum: 260.5 mg AA Eq./g CUPRAC: 662.7 mg Trolox Eq./g FRAP: 168.7 mg Trolox Eq./g β-carotene bleaching: 58.2%–84.3% Metal chelating: 2.9%–41.6%	[84]
	Boraginaceae	*Arnebia densiflora*	Root	Not specified	DPPH: 9.2%–56.2% Iron chelating: 7.7%–56.5%	[85]
		*Onosma nigricaule*	Root	Phenolics, Flavonoids	NO: 42.7% Iron chelating: 3.1%	[86]
	Cistaceae	*Cistus laurifolius*	Leaves	Phenolics, Flavonoids	DPPH: 14.3%–87.8% FRAP: 0.1–3.0 abs.	[87]
	Hypericaceae	*Hypericum capitatum*	Aerial parts	Not specified	DPPH: 83.2% DMPD: 28.5% NO: 3.4%	[86]
	Lamiaceae	*Ballota nigra*	Whole plant	Palmitic acid, Linoleic acid, Oleic acid, Linolenic acid, Stearic acid, Phytol, Arachidic acid, Behenic acid, 11,13-Dimethyl-12-tetradecen-1-ol acetate, Myristic acid, 10-Undecenoic acid, 7-Methylhexadecenoic acid, Palmitoleic acid	ABTS: ≈3.0%–88.0% CUPRAC: 0.0–1.1 abs.	[88]
		*Origanum vulgare*	Essential oil	Linalool, Thymol, Carvacrol, *p*-Cymene, γ-Terpiene, β-Caryophyllene, α-Terpiene, Borneol, 1-Octen-3-ol, Caryophyllene oxide, α-Thujene, cis-Linalol oxide, *trans*-Linalol oxide, α-Pinene, β-Bisabolene, *p*-Cymene-8-ol, Elemol, α-Terpineol, Camphene, Hotrienol, α-Humulene	DPPH: 0.0–57.2 mg Trolox Eq./g oil ABTS: 9.6–176.4 mg Trolox Eq./g oil FRAP: 17.1–133.3 mg Trolox Eq./g oil CUPRAC: 46.6–222.1 mg Trolox Eq./g oil β-carotene bleaching: 24.0%–99.9% Phosphomolybdenum: 0.8–8.1 mmol Trolox Eq./g oil Metal chelating: 1.3-3.8 mg EDTA Eq./g oil	[62]
		*Salvia fruticosa*	Aerial parts	Rosmarinic acid	DPPH: 88.2%	[89]
		*Salvia trichoclada*	Aerial parts	Rosmarinic acid	DPPH: 89.6%	[89]
		*Salvia verticillata*	Aerial parts	Rosmarinic acid	DPPH: 86.1%	[89]
		*Sideritis galatica*	Essential oil	β-Pinene, α-Pinene, β-Caryophyllene, (*Z*)-β-Ocimene, Limonene, Benzyl benzoate, Sabinene, β-Phellandrene, (*E*)-β-Ocimene, Germacrene D, Caryophyllene oxide, Bicyclogermacrene, δ-3-Carene, *p*-Cymene, (*Z*)-β-Farnesene, α-Phellandrene, Terpinolene, Heptanal, α-Humulene, 1-Octen-3-ol, δ-Cardinene, α-Copaene, (*E*)-2-Hexenal	DPPH (IC_50_): 16.5 mg/mL ABTS (IC_50_): 8.5 mg/mL NO (IC_50_): 0.9 mg/mL CUPRAC (EC_50_): 1.1 mg/mL FRAP (EC_50_): 2.1 mg/mL Phosphomolybdenum: 2.6 mg Trolox Eq./g oil Metal chelating: 29.1 mg EDTA Eq./g oil	[63]
	Myrtaceae	*Myrtus communis*	Leaf, fruit	Phenolics, Flavonoids	FRAP: 0.3–3.4 abs Phosphomolybdenum: 0.3–0.5 abs DPPH: >90% DMPD: 40.1%–44.1% Metal chelating: 79.3%	[90]
	Rosaceae	*Potentilla anatolica*	Aerial parts	Phenolics, Flavonoids, Saponins, Triterpenoids	DPPH: 302.8–334.7 mg Trolox Eq./g ABTS: 4.9–5.2 mmol Trolox Eq./g Phosphomolybdenum: 3.3–4.8 mmol Trolox Eq./g FRAP: 223.6–233.0 mg Trolox Eq./g CUPRAC: 291.7–340.8 mg Trolox Eq./g Metal chelating: 27.4–32.9 mg EDTA Eq./g	[66]
	Rubiaceae	*Galium spurium*	Aerial parts	Deacetyl-asperulosidic acid, Monotropein, Asperulosidic acid, Isoorientin, Rutin, Isoquercitrin, Quercetin, Chlorogenic acid, Caffeic acid, Ursolic acid	Not specified	[91]
	Rutaceae	*Haplophyllum myrtifolium*	Aerial parts	Phenolics, Flavonoids, Tannins, Saponins, Flavanols	DPPH: 43.8–84.5 mg Trolox Eq./g ABTS: 129.3–263.5 mg Trolox Eq./g NO: 2.6–7.0 mmol Trolox Eq./g Phosphomolybdenum: 1.7–3.3 mmol Trolox Eq./g Metal chelating: 8.4–41.8 mg EDTA Eq./g FRAP: 0.4–0.7 mmol Trolox Eq./g CUPRAC: 0.5–0.8 mmol Trolox Eq./g	[67]
	Zingiberaceae	*Alpinia officinarum*	Rhizomes	Pyrogallol, Kaempferol, Apigenin, Isorhamnetin, *p*-OH benzoic acid, *p*-Coumaric acid, Quercetin, Luteolin	Fe^3+^ reducing (IC_50_): 0.7–1.2 μg/mL CUPRAC (IC_50_): 0.6–1.2 μg/mL FRAP (IC_50_): 1.3–2.0 μg/mL Fe^2+^ chelating (IC_50_): 8.6–12.4 μg/mL DPPH (IC_50_): 14.7–49.5 μg/mL ABTS (IC_50_): 12.4–33.0 μg/mL	[92]
Gastric Ulcers	Altingiaceae	*Liquidambar orientalis*	Bark	Vinyl benzene, Cinnamyl alcohol, (*E*)-Methyl cinnamate, Benzene propanol, α-Pinene, (*E*)-Cinnamaldehyde, β-Pinene, 1,8-Cineole, 4-Ethyl phenol, *p*-Cymene, Benzaldehyde, 2-Ethyl hexanol, (*Z*)-Methyl cinnamate, Limonene, Linalool, δ-Cadinol, α-Terpineol, β-Elemene, Acetophenone, γ-Terpinene, Hexadecane, Terpinen-4-ol, Borneol, α-Terpinyl acetate, Camphene, Pentadecane, Bornylacetate, α-Muurolene, Benzene propanal, Benzylalcohol, 4-Ethylguaiacol, (*E*)-Ethylcinnamate, Cinnamylacetate	Not specified	[93]
	Calamitaceae	*Equisetum palustre* L.	Aerial parts	Kaempferol-3-*O*-1’’-β-d-glucopyranosyl-3-*O*-1’’-β-d-glucopyranoside	Not specified	[94]
	Caprifoliaceae	*Sambucus ebulus* L.	Aerial parts	Quercetin-3-*O*-monoglucoside, Isorhamnetin-3-*O*-monoglucoside	Not specified	[95]

AA: ascorbic acid; ABTS: 2,2-azinobis 3-ethylbenzothiazoline-6-sulfonic acid diammonium salt; CAT: catalase; CUPRAC: cupric ion reducing antioxidant capacity; DMPD: *N*,*N*-dimethyl-*p*-phenylendiamine; DPPH: 1,1-diphenyl-2-picrylhydrazyl; FRAP: ferric reducing antioxidant power; GR: glutathione reductase; GSH: reduced glutathione; GSH-Px: glutathione peroxidase; GST: glutathione-*S*-transferase; MDA: malondialdehyde; NO: nitric oxide, ORAC: oxygen radical absorbance capacity; SOD: superoxide dismutase; TBARS: thiobarbituric acid reactive substances.
